# The Draft Genome Sequence of *Sphingomonas paucimobilis* Strain HER1398 (*Proteobacteria*), Host to the Giant PAU Phage, Indicates That It Is a Member of the Genus *Sphingobacterium* (*Bacteroidetes*)

**DOI:** 10.1128/genomeA.00598-13

**Published:** 2013-08-08

**Authors:** Richard Allen White, Curtis A. Suttle

**Affiliations:** Department of Microbiology & Immunology, University of British Columbia, Vancouver, British Columbia, Canadaa; Department of Botany, University of British Columbia, Vancouver, British Columbia, Canadab; Department of Earth, Ocean & Atmospheric Sciences, University of British Columbia, Vancouver, British Columbia, Canadac; Canadian Institute for Advanced Researchd ‡

## Abstract

The draft genome sequence of *Sphingomonas paucimobilis* host index number (HER) 1398, host of the giant PAU phage isolated from silk moths (*Bombyx mori*), indicates that this isolate belongs within the genus *Sphingobacterium*. We suggest that *Sphingomonas paucimobilis* strain HER1398 be reclassified as *Sphingobacterium paucimobilis* strain HER1398.

## GENOME ANNOUNCEMENT

*Sphingomonas paucimobilis* host index number (HER) 1398 was obtained from the Félix D’Hérelle Reference Center for Bacterial Viruses at the University of Laval, Quebec City, Canada. It was isolated by S. Basavarajappa and C. Savanurmath from a homogenate of diseased silkworms (*Bombyx mori*) collected from various areas within Karnataka State, India, and is a host for the giant phage, PAU (1). The isolate was originally classified as *Pseudomonas paucimobilis* (1), but it was reclassified as *Sphingomonas paucimobilis* strain HER1398 by the Félix D’Hérelle Reference Center.

The draft genome sequence indicates that *Sphingomonas paucimobilis* HER1398 is a member of the genus *Sphingobacterium*. BLAST analysis indicates 94 to 96% identity of the 16S rRNA genes to other members of this genus, including *Sphingobacterium bambusae* and *Sphingobacterium composti* (2, 3). Moreover, *Sphingomonas* spp. and *Sphingobacterium* are members of different phyla, *Proteobacteria* and *Bacteroidetes*, respectively.

High-quality DNA was extracted using Qiagen QiaAMP followed by MinElute Cleanup columns. The Illumina library was constructed using Lucigen’s NxSeq library prep kit without final PCR enrichment. Quality control of the resulting library was completed using Agilent high-sensitivity DNA chips and digital droplet PCR (4).

Whole-genome shotgun sequencing was completed using Illumina MiSeq and HiSeq in the 250-bp and 100-bp paired-read formats, respectively. A partial flow cell of HiSeq/MiSeq obtained 2.86 million raw paired reads and 626,550,788 bp of raw sequence. The reads were merged using FLASH (version 1.2.5) screened for phiX using Bowtie2 (version 2.1.0), then filtered using Picard tools (version 1.90) (http://picard.sourceforge.net/) (5, 6). Reads were assembled using Ray assembler (version 2.2.0), yielding 23 contigs summing to 5,126,359 bp, with an average contig length of 222,885 bp, with the largest contig being 1,001,915 bp, (*N*_50_ length, 416,944 bp; *N*_90_ length, 132,253 bp; G+C content, 40.86%) (7, 8).

Annotation of the draft genome was conducted on the RAST annotation server using the Glimmer-3 option FigFam database version-59 (9). The genome annotation contains 4,475 predicted protein-coding genes, including 79 noncoding RNAs and 371 predicted SEED subsystem features. Given its role as the host for phage PAU, the genome was screened for evidence of phages, prophages, gene-transfer agents (GTAs), CRISPR arrays, and other phage-resistance elements (such as exopolysaccharide [EPS]) or abortive infection systems (10); none were found. However, type I and type III restriction-modification systems were predicted, which could help avoid phage infection (10). In addition, there are predicted genes for antibiotic resistance to aminoglycosides, fluoroquinolones, and beta-lactams. The resistance to these antibiotics could be due to predicted aminoglycoside adenylyltransferases, beta-lactamases, and DNA gyrases. We found *S. paucimobilis* HER1398 to be resistant to tobramycin (10 µg), an aminoglycoside, and penicillin (10 IU), a beta-lactam. Fluroquinolone resistance has not been tested.

Based on analysis of the full-length 16S rRNA gene sequence and whole-genome shotgun sequencing, it is clear that *Sphingomonas paucimobilis* HER1398 has been incorrectly classified. Based on sequence comparison it should be classified as *Sphingobacterium paucimobilis* HER1398, a member of a different phylum. This is the fourth *Sphingobacterium* genome sequenced, but the first isolated from an insect.

### Nucleotide sequence accession numbers.

This whole-genome shotgun project has been deposited at DDBJ/EMBL/GenBank under the accession number ATDL00000000. The version described in this paper is version ATDL01000000.

## References

[1] Ackermann HW Auclair P Basavarajappa S Konjin HP Savanurmath C 1994 Bacteriophages from Bombyx mori. Arch. Virol. 137:185–190 7979992 10.1007/BF01311186

[2] Duan S Liu Z Feng X Zheng K Cheng L 2009 *Sphingobacterium bambusae* sp. nov., isolated from soil of bamboo plantation. J. Microbiol. 47:693–698 20127461 10.1007/s12275-009-0296-2

[3] Ten LN Liu QM Im WT Aslam Z Lee ST 2006 *Sphingobacterium composti* sp. nov., a novel DNase-producing bacterium isolated from compost. J. Microbiol. Biotechnol. 16:1728–1733

[4] Hindson BJ Ness KD Masquelier DA Belgrader P Heredia NJ Makarewicz AJ Bright IJ Lucero MY Hiddessen AL Legler TC Kitano TK Hodel MR Petersen JF Wyatt PW Steenblock ER Shah PH Bousse LJ Troup CB Mellen JC Wittmann DK Erndt NG Cauley TH Koehler RT So AP Dube S Rose KA Montesclaros L Wang S Stumbo DP Hodges SP Romine S Milanovich FP White HE Regan JF Karlin-Neumann GA Hindson CM Saxonov S Colston BW 2011 High-throughput droplet digital PCR system for absolute quantitation of DNA copy number. Anal. Chem. 83:8604–8610 22035192 10.1021/ac202028g PMC3216358

[5] Magoč T Salzberg SL 2011 FLASH: fast length adjustment of short reads to improve genome assemblies. Bioinformatics 27:2957–2963 21903629 10.1093/bioinformatics/btr507 PMC3198573

[6] Langmead B Salzberg S 2012 Fast gapped-read alignment with Bowtie 2. Nat. Methods 9:357–359 22388286 10.1038/nmeth.1923 PMC3322381

[7] Boisvert S Laviolette F Corbeil J 2010 Ray: simultaneous assembly of reads from a mix of high-throughput sequencing technologies. J. Comput. Biol. 11:1519–1533 20958248 10.1089/cmb.2009.0238 PMC3119603

[8] Boisvert S Raymond F Godzaridis E Laviolette F Corbeil J 2012 Ray Meta: scalable de novo metagenome assembly and profiling. Genome Biol. 13:R122. 10.1186/gb-2012-13-12-r122 23259615 PMC4056372

[9] Aziz RK Bartels D Best AA DeJongh M Disz T Edwards RA Formsma K Gerdes S Glass EM Kubal M Meyer F Olsen GJ Olson R Osterman AL Overbeek RA McNeil LK Paarmann D Paczian T Parrello B Pusch GD Reich C Stevens R Vassieva O Vonstein V Wilke A Zagnitko O 2008 The RAST server: rapid annotations using subsystems technology. BMC Genomics 9:75. 10.1186/1471-2164-9-75 18261238 PMC2265698

[10] Labrie SJ Samson JE Moineau S 2010 Bacteriophage resistance mechanisms. Nat. Rev. Microbiol. 8:317–327 20348932 10.1038/nrmicro2315

